# Is endoscopic treatment beneficial in patients with clinically suspicious of common bile duct stones but no obvious filling defects during the ERCP examination?

**DOI:** 10.1186/s12876-016-0524-2

**Published:** 2016-08-26

**Authors:** Po-Hung Chiang, Kwok-Hung Lai, Tzung-Jiun Tsai, Kung-Hung Lin, Kai-Ming Wang, Sung-Shuo Kao, Wei-Chih Sun, Jin-Shiung Cheng, Ping-I Hsu, Wei-Lun Tsai, Wen-Chi Chen, Yun-Da Li, E-Ming Wang, Huey-Shyan Lin, Hoi-Hung Chan

**Affiliations:** 1 Division of Gastroenterology, Department of Internal Medicine, Kaohsiung Veterans General Hospital, 386 Ta-Chung 1st Road, Kaohsiung, 81362 Taiwan; 2 School of Medicine, National Yang-Ming University, No. 155, Sec. 2, Li-Nong Street, Pei-Tou, Taipei, 112 Taiwan; 3 Department of Health-Business Administration, Fooyin University, 151 Jinxue Rd, Daliao Dist, Kaohsiung City, 83102 Taiwan; 4 Department of Biological Sciences, National Sun Yat-sen University, 70 Lien-Hai Road, Kaohsiung, 80424 Taiwan; 5 Department of Business Management, National Sun Yat-sen University, 70 Lien-Hai Road, Kaohsiung, 80424 Taiwan; 6 College of Pharmacy and Health Care, Tajen University, 20 Weisin Road, Sin-er Village, Yanpu Township, Pingtung County 907 Taiwan

**Keywords:** ERC, ERCP, CBDS

## Abstract

**Background:**

Sometimes, no definite filling defect could be found by cholangiogram (ERC) during the endoscopic retrograde cholangio-pancreatiographic (ERCP) exam; even prior images had evidence of common bile duct stones (CBDS). We aimed in estimating the positive rate of extraction of CBDS who had treated by endoscopic sphincterotomy/endoscopic papillary balloon dilation (EST/EPBD) with negative ERC finding.

**Methods:**

One hundred forty-one patients with clinically suspicious of CBDS but negative ERC, who had received EST/EPBD treatments was enrolled. Potential factors for predicting CBDS, as well as the treatment-related complications were analyzed.

**Results:**

Nearly half of the patients with negative ERC, had a positive stone extraction. Only patients with high probability of CBDS were significantly associated with positive stone extraction. Moreover, patients with intermediate probability of CBDS had higher rates of overall complications, including post-ERCP pancreatitis. In addition, no significant difference of post-ERCP pancreatitis was found between EST and EPBD groups in any one group of patients with the same probability of CBDS.

**Conclusions:**

Regarding patients with negative ERC, therapeutic ERCP is beneficial and safe for patients present with high probability of CBDS. Moreover, under the same probability of CBDS, there was no significance difference in post-ERCP pancreatitis between EST and EPBD.

## Background

Common bile duct stone (CBDS) is an important clinical problem that can cause serious complications, such as acute cholangitis and pancreatitis [1]. Therefore, it is recommended to remove the stones endoscopically or surgically once diagnosis is established [2]. However, sometimes, early definitive diagnosis of choledocholithiasis is difficult and should be based on clinical symptoms and signs, biochemical data and image findings.

Persist elevation of serum alkaline phosphatase (ALP) and alanine transaminase (ALT) were shown to correlate with the presence of CBDS even with a normal-sized CBD [3]. A recent study showed that trans-abdominal ultrasound alone is inadequate to predict the CBDS in patients presenting with acute cholecystitis [4].

Endoscopic retrograde cholangiopancreatography (ERCP) is generally believed to be the gold standard for both diagnosis and treatment of CBDS. However, inevitably, the procedure is associated with an overall complication rate of 4 ~ 10 % and mortality rate of 0.02 ~ 0.5 % [5–10]. The major complications include pancreatitis (1.3 ~ 6.7 %), infection (0.3 ~ 5.0 %), hemorrhage (0.3 ~ 2.0 %), and perforation (0.1 ~ 1.1 %) [6, 9, 11]. Others include cardiac (<0.1 %), and pulmonary events (<0.1 %) [6]. Therefore, currently, purely diagnostic ERCP is not suggested [7, 8]. Instead, relative non-invasive imaging modalities such as MRCP and EUS are preferred.

**Fig. 1:**
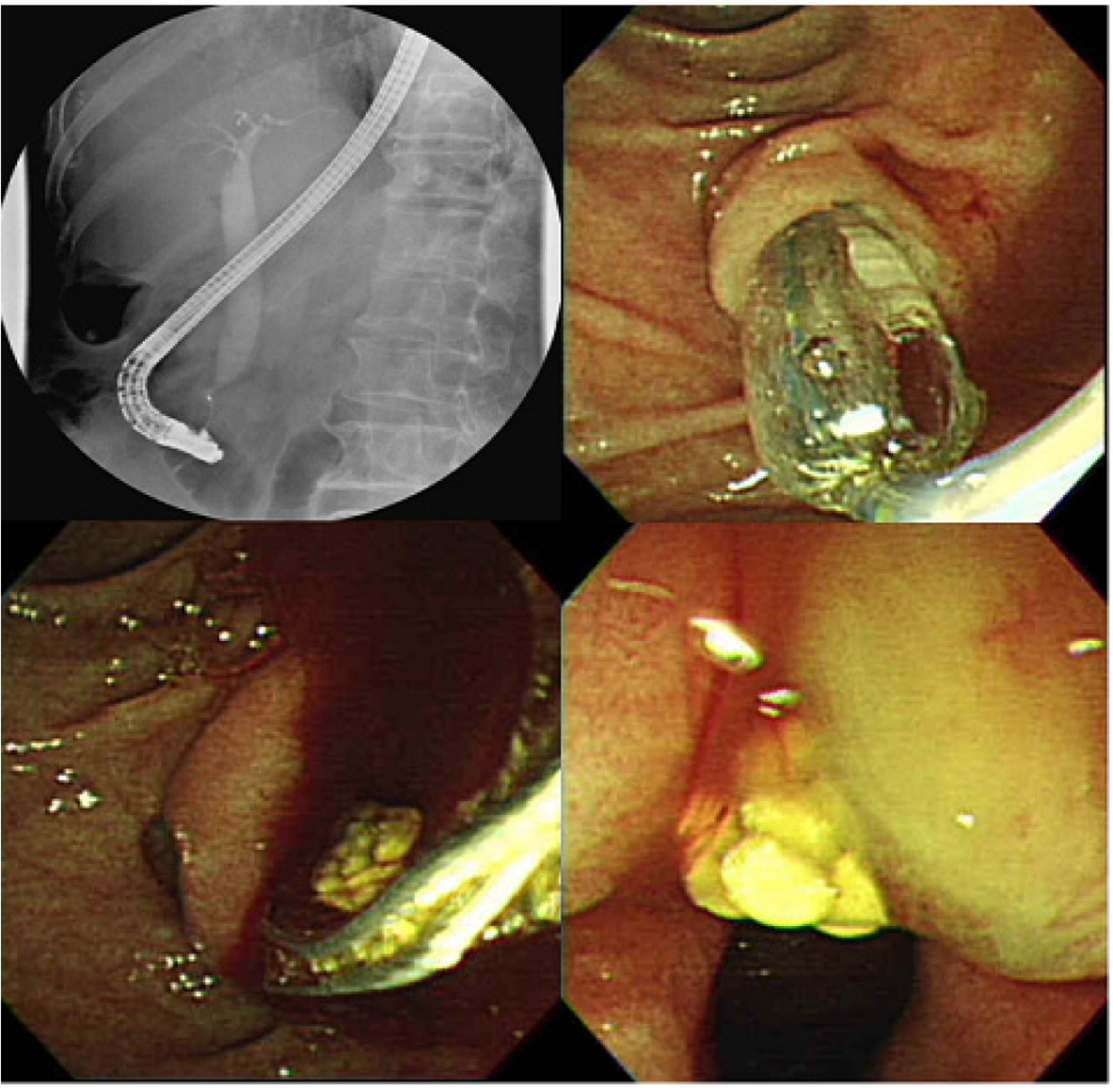
A female patient presented with epigastric pain, jaundice, while CT scan showing dilated CBD and suspicious of CBDS. In addition, no definite filling defect was found by ERC. However, EPBD was performed and a 1-cm hard stone was extracted subsequently

In 2010, the American Society for Gastrointestinal Endoscopy (ASGE) established a general rule for the evaluation of likelihood of choledocholithiasis, in which; Patients were divided into “high probability (risk of CBDS > 50 %)”, “intermediate probability (risk of CBDS: 10 ~ 50 %)”, and “low probability (risk of CBDS < 10 %)” groups [12]. In addition, the author also pointed-out the management algorithm for patients with symptomatic choledocholithiasis [12]. However, sometimes, no obvious filling defects inside CBD could be found by cholangiogram (ERC, Fig. 1), even prior images, such as trans-abdominal ultrasound or CT scan, had demonstrated the evidence of CBDS. Regarding the possible complications, further the treatment procedures, such as endoscopic sphincterotomy (EST) and/or papillary balloon dilation (EPBD) in this situation is worthy consideration. The aim of this retrospective study was to estimate the positive rate of CBDS in patients with negative filling defects from ERC, and the factors for possible CBDS prediction, as well as the treatment-related complications (safety concern).

## Methods

### Study design, definition and patient selection

This retrospective study was approved by Institutional Review Board of Kaohsiung Veterans General Hospital. Eligibility of patients includes those who were clinically classified as either intermediate or high risks for CBDS [12] according to symptoms and signs, laboratory data or image studies during the period of April 2008 to March 2014. These patients had received either EST or EPBD treatments, although no obvious filling defect was detected by ERC. Exclusion criteria include peri-ampullary tumors, hepatocellular disease, hemolytic disease, and patients who ever received endoscopic or surgical treatment for bile duct stones. In addition, positive extraction of stones was defined as stones detectable by naked eyes through the video endoscopic pictures during extracting the bile duct by using basket or balloon catheter; or presence of microlithiasis (non-visible by naked eyes) under microscopic exams of the bile. The model of “probability of CBDS” from American society for gastrointestinal endoscopy (ASGE, 2010) was applied in this study, in which high probability of CBDS includes: 1) CBDS seen on trans-abdominal ultrasonography (US) (and/or CT scan), 2) signs of acute cholangitis (people who had Charcot’s triad), 3) total bilirubin > 4 mg/dL, 4) both dilated CBD on US (>6 mm with gallbladder in situ and > 10 mm with cholecystectomy) and total bilirubin level 1.8 ~ 4 mg/dL; and intermediate probability of CBDS includes: 1) either one of these two factors: dilated CBD on US or total bilirubin level 1.8 ~ 4 mg/dL, 2) advanced age (>55 year-old), 3) elevation of a liver biochemical test other than bilirubin, and 4) gallstone pancreatitis. In addition, ERCP-related complications were defined and graded in severity according to the consensus criteria, which was adapted as (Table 1), developed by Cotton et al. [6, 7, 11].

**Table 1:** Consensus criteria for ERCP complications^ab^

	Mild	Moderate	Severe
Bleeding	Clinical evidence of bleeding (ie, not just endoscopic); Hb level drop <3 g; no need for transfusion.	Transfusion: ≤4 units; no angiographic intervention or surgery.	Transfusion: ≥5 units or intervention (angiographic or surgical).
Perforation	Possible, or only very slight leak of fluid or contrast dye; treatable by fluids and suction for ≤3 days.	Any definite perforation treated medically for 4–10 days.	Medical treatment for more than 10 days or intervention (percutaneous or surgical).
Pancreatitis	Clinical pancreatitis; amylase at least 3 times normal at more than 24 hours after the procedure requiring admission or prolongation of planned admission to 2–3 days.	Pancreatitis requiring hospitalization of 4–10 days.	Pancreatitis requiring hospitalization for more than 10 days, or hemorrhagic pancreatitis, phlegmon or pseudocyst, or intervention (percutaneous drainage or surgery).
Infection (cholangitis)	>38°C at 24–48 hours.	Febrile or septic illness requiring >3 days of hospital treatment or endoscopic or percutaneous intervention	Septic shock or surgery.

ie, mild, unplanned hospital stay of 2–3 nights; moderate, 4–10 nights; and severe (>10 nights or intensive care or surgery)

^a^From Ref. 6 and 11. ERCP, endoscopic retrograde cholangiopancreatography

^b^All other complications were graded for severity of the need for hospitalization and/or surgical treatment

#### Endoscopic Procedures

Patients were conscious for the procedure and received 10 % xylocaine spray for local anesthesia of the pharynx, intramuscular injection with 40 mg hyoscine-*N*-butylbromide, and intramuscular injection with 25–50 mg meperidine. ERCP was performed in the standard manner using a side-view endoscope (JF-240; Olympus Optical Corporation, Tokyo, Japan). After selective cannulation of the common bile duct by the catheter, cholangiography was performed to evaluate the presence/absence of filling defects inside CBD. A 0.035-in. guide wire was then inserted into the bile duct through the catheter. For EST group, sphincterotomy was done by using a wire-guided sphincterotome. Incision was started at the orifice of papilla and extended upward to the direction of bile duct. For EBPD, selective cannulation of the common bile duct with guide wire insertion was the same as EST. A dilating balloon (CRE balloon; Boston Scientific, Corp, Ireland) was passed via the prepositioned 0.035-in. guide wire into the bile duct. Using fluoroscopic (AXIOM, Iconos R200, Siemens AG 2002) and endoscopic guidance, the balloon was inflated with sterile saline solution up to the optimal size (at least > 6 mm in diameter) and duration (from 1.5 to 5 min) according to the patients’ condition and tolerance. In order to minimize the risk of perforation, the size of the balloon should be not exceed the size of the CBD. After the balloon and guide wire were removed, the CBDS was retrieved out using a Dormia basket or balloon-tipped catheter with or without the aid of mechanical lithotripsy (BML-4Q; Olympus Optical, Tokyo, Japan). Unnecessary cannulation or contrast injection of pancreatic duct was avoided.

### Statistical analysis

All statistical analyses were performed using the PASW 20.0 (IBM, New York, NY, USA). Continuous valuables are expressed as mean ± SD. Chi-square analyses or Fisher’s exact tests were used for comparing categorical variables, while independent t-tests were used for comparing continuous variables between patients with final positive and negative stone extraction. Associations between the possible predictors and the positivity of CBD stones and between the possible predictors and complications were assessed by multiple logistic regressions. Results were shown as odds ratios and 95 % confidence intervals (CIs). A *p*-value less than 0.05 was considered statistically significant.

## Results

Demographic data was shown in Table 2. No significant difference was found at gender, age, body mass index (BMI), initial GPT and ALP level, history of cholecystectomy, presence/absence of gallbladder stones, and juxta-papillary diverticulum (JPD), between patients with final positive or negative stone extraction. There were only initial cholangitis and high probability of CBDS significantly associated with positive stone extraction. There were total 141 (male/female: 81/60) patients, clinically suspicious of CBD stones (intermediate probability: 28, high probability: 113), undergoing successful therapeutic ERCP (EST/EPBD: 30/111) with which pre-treatment cholangiogram (ERC) showed no obvious filling defects. For the group of positive stone extraction (70 patients), there were 64 patients showed detectable (all are barely visible by naked eyes and un-measurable) stones and six patients showed microlithiasis under microscopic analysis of bile. However, there were only 21 samples of bile available for analysis (21/141 = 14.9 %). With regard to the high probability group of CBDS, 65 cases presented with evidence of CBDS at initial image, 25 with acute cholangitis, 10 with total bilirubin level >4 mg/dL, and 13 with mild elevated total bilirubin (1.8–4 mg/dL) and CBDdilatation. On the other hand, in the intermediate probability group, 16 cases presented with mild elevated total bilirubin (1.8–4 mg/dL) without CBD dilatation, six with CBD dilatation without elevated total bilirubin, two with gallstone pancreatitis, and four with age > 55 year-old. Besides, the mean length of EST was 0.91 cm (0.5 cm ~ 1.5 cm); and the mean size of dilating balloon was 0.99 cm (0.6 cm ~ 1.8 cm), depend on the relative sizes of CBD. ERCP was performed at a mean of four days after admission.

**Table 2:** Demographic data between groups with and without stone extraction

Characteristics	Stone (+)	Stone (−)	*P-value*
	(*n* = 70)	(*n* = 71)	
Gender (Male/Female)	40/30	41/30	0.942
Age	66.73 ± 17.93	61.63 ± 15.55	0.073
BMI	24.54 ± 3.58	24.77 ± 3.95	0.736
Cholecystectomy	8 (11.4 %)	8 (11.3 %)	0.976
GB stone	54 (77.1 %)	54 (76.1 %)	0.879
JPD	31 (44.3 %)	26 (36.6 %)	0.354
ALT at admission	278.9 ± 282.8	283.7 ± 260.1	0.917
Alk-P at admission	175.1 ± 120.7	162.5 ± 149.7	0.590
Total bilirubin at admission	3.49 ± 2.37	3.35 ± 2.20	0.726
CBDS risk (high vs. intermediate)	62 (88.6 %)	51 (71.8 %)	0.013*
Cholangitis	29 (41.4 %)	14 (19.7 %)	0.005*
Pancreatitis	26 (37.1 %)	22 (31.0 %)	0.440

*Abbreviations*: *BMI* body mass index, *GB* gallbladder, *JPD* juxta-papillary diverticulum, *ALT* aspartate transaminase, *Alk-P* alkaline phosphatase; CBDS, common bile duct stone

*p < 0.05

By using multiple logistic regressions, only high probability of CBDS was found to be significantly associated with positive stone extraction (high vs. intermediate probability: 54.9 % vs. 28.6 %, *p* = 0.039) (Table 3). Moreover, there were totally 11 (7.8 %) complications found in the study (Table 4). By using multiple logistic regressions, intermediate probability of CBDS was associated with higher risk of overall complications and post-ERCP pancreatitis (*p* = 0.043; *p* = 0.007) (Tables 4 and 5). In addition, no significant difference in overall complications, including post-ERCP pancreatitis, was found between EST and EPBD groups under the same probability of CBDS, no matter high or intermediate probability. There were three (mild/moderate/severe: 1/1/1) and four (mild/moderate/severe: 1/3/0) post-ERCP pancreatitis found in EST and EPBD groups, respectively. Moreover, two mild cholangitis combined with moderate pancreatitis and two pure cholangitis (mild/moderate/severe: 1/0/1) were found in EPBD group. However, no procedure-related mortality was noted in the current study.

**Table 3:** Risk factors of patients with stone extraction

Characteristics	Complication (+)	Complication (−)	*P-value*	*OR*	*95 % CI of OR*
	(*n* = 70)	(*n* = 71)			
CBDS probability from ASGE					
CBDS risk (high vs. intermediate)	62 (88.6 %)	51 (71.8 %)	0.039*	2.670	1.050 ~ 6.790
Characteristics					
Gender (M/F)	40/30	41/30	0.992	1.004	0.496 ~ 2.031
BMI	24.54 ± 3.58	24.77 ± 3.95	0.540	0.971	0.883 ~ 1.067
Cholecystectomy	8 (11.4 %)	8 (11.3 %)	0.634	1.430	0.329 ~ 6.225
GB stone	54 (77.1 %)	54 (76.1 %)	0.504	1.453	0.486 ~ 4.349
JPD	31 (44.3 %)	26 (36.6 %)	0.212	1.593	0.767 ~ 3.310

*Abbreviation*: *OR* odd’s ratio

*p < 0.05

**Table 4:** Risk factors of the patients with complication

Characteristics	Complication (+)	Complication (−)	*P-value*	*OR*	*95 % CI of OR*
	(*n* = 11)	(*n* = 130)			
Procedure					
EST/EPBD	3 (27.3 %)	27 (20.8 %)	0.909	0.919	0.215 ~ 3.920
CBDS probability from ASGE					
CBDS risk (high vs. intermediate)	6 (54.5 %)	107 (82.3 %)	0.043*	0.262	0.072 ~ 0.958

*Abbreviations*: *EST* endoscopic sphincterotomy, *EPBD* endoscopic papillary balloon dilation

*p < 0.05

**Table 5:** Risk factors of the patients with post-ERCP pancreatitis

Characteristics	Post-ERCP pancreatitis (+)	Post-ERCP pancreatitis (−)	*P-value*	*OR*	*95 % CI of OR*
	(*n* = 7)	(*n* = 134)			
Procedure					
EST/EPBD	3 (42.9 %)	27 (20.1 %)	0.442	0.523	0.100 ~ 2.735
CBDS probability from ASGE					
CBDS risk (high vs. intermediate)	2 (28.6 %)	111 (82.8 %)	0.007*	0.093	0.017 ~ 0.523

*p < 0.01; correlation between procedure and CBDS probability from ASGE showed no significance

*(p = 0.456)*

## Discussion

According to the current study, nearly half (49.6 %) of patients without detected filling defects in ERC, have evidence of positive stone extraction after EST or EPBD treatments. By multiple logistic regressions, only high probability of CBDS was significantly associated with positive stone extraction.

Total complication rate among patients received EST or EPBD with negative filling defects from ERC was 7.80 %, and no significant difference was found between these two treatment modalities. In addition, there was no procedure-related mortality. Furthermore, intermediate probability of CBDS was associated with higher risk of overall complications, including post-ERCP pancreatitis. Therefore, endoscopic treatment (EST or EPBD) is beneficial and safe for patients with high probability of CBDS. In addition, no significant difference in overall complications, as well as post-ERCP pancreatitis, was found between EST and EPBD groups under the same probabilities of stones.

The lack of important roles of liver function tests, such as GPT, ALP before ERCP in the current results, as in the previous studies [13–16] might be due to the small sample size.

Moreover, bile analysis was inadequately done in this study (14.9 %). Therefore, prospective study with bile analysis of microlithiasis is crucial to elucidate the true rate of CBDS in patients with negative filling defects in ERC. In addition, endoscopic ultrasound might be done before EST and EPBD in order to minimize the ERCP-associated complications and to quickly delineate the presence of small stones or sludge in the CBD [5].

## Conclusions

The probability of CBDS (high vs. intermediate probability) could play a significant role in the estimation of positive stone extraction before deciding the therapeutic strategies, with the result in fewer overall complications, including post-ERCP pancreatitis after the treatment even though the negative filling defect on ERC. In addition, endoscopic treatment (EST or EPBD) is beneficial and safe to patients with high probability of CBDS. Moreover, under the same probability scores, there was no significant difference in post-ERCP pancreatitis between EST and EPBD. Future prospective study with bile analysis of microlithiasis is important to elucidate the true rate of CBDS in patients with negative filling defects in ERC.

## Abbreviations

CBDS: common bile duct stones
EPBD: endoscopic balloon dilation
ERC: endoscopic retrograde cholangiography
ERCP: endoscopic retrograde cholangio-pancreatography
EST: endoscopic sphincterotomy
